# Injury risk and epidemiology of pickleball players in South Korea: a cross-sectional study

**DOI:** 10.3389/fpubh.2025.1617291

**Published:** 2025-08-11

**Authors:** Boseok Jeong, Kang-Jun Lee, Seung-Hee Nam, Sua Im, Rokbit S. Lee, Jinmoo Heo, Kyung-Min Kim

**Affiliations:** ^1^Department of Sport Science, Sungkyunkwan University, Suwon-si, Republic of Korea; ^2^Department of Sport Industry Studies, Yonsei University, Seoul, Republic of Korea

**Keywords:** racket sports, sports injury, injury prevalence, sports safety, epidemiology, pickleball

## Abstract

**Background:**

Pickleball is rapidly growing in popularity, yet limited research exists regarding injury epidemiology and associated risk factors, particularly in South Korea. This study aimed to investigate the prevalence, characteristics, and predictors of injuries among recreational pickleball players.

**Methods:**

A cross-sectional survey was conducted during the 1st HEAD Korea Open Pickleball Championship in 2024, with 232 participants (mean age 50.5 ± 12.2 years) completing a questionnaire on demographics, playing habits, and injuries experienced over the previous 12 months.

**Results:**

34.2% reported at least one injury. The most affected regions were the knee (23.3%), elbow or forearm (18.1%), and shoulder or upper arm (17.2%). Overuse injuries (i.e., those with a gradual onset and no single traumatic event) accounted for 78% of reported cases, while 22% were related to acute trauma. The most frequent injury types were muscle or tendon injuries (33.3%) and joint sprains or dislocations (28.3%). Logistic regression analysis identified higher self-rated skill level as significantly associated with a reduced risk of injury (odds ratio [OR] = 0.789, 95% confidence interval [CI]: 0.624–0.992, *p* = 0.044), as well as greater weekly play hours (OR = 0.913, 95% CI: 0.861–0.963, *p* = 0.001). In contrast, age, gender, total playing experience, and frequency of weekly play were not significantly associated with injury risk.

**Conclusion:**

These findings underscore the need for skill-based and volume-conscious injury prevention strategies, particularly for beginner and recreational players, to support safe participation in this fast-growing sport.

## 1 Introduction

Pickleball, a sport combining elements of badminton, tennis, and table tennis, has experienced a significant rise in popularity worldwide, especially among older adults. Since its invention in the 1960s, pickleball has evolved from a simple backyard game to an organized competitive sport that attracts players of all ages and abilities (1). Its rapid growth can be attributed to factors such as its short learning curve, relatively low cost, and the emphasis on community engagement and health (2). In the United States, pickleball is widely considered the fastest growing sport, representing an increase of approximately 85% in the number of players between 2022 and 2023, reaching nearly 8.9 million players in the United States (1, 3). Similarly, its popularity is rising in South Korea, reflected in major tournaments like the Korea Open Pickleball Championship, which had 344 participants, and the World Pickleball Championship Korea, an international competition in Asia that included 654 registered teams and 249 individual players in 2024 (4).

As the popularity of pickleball continues to grow, research into various aspects of pickleball has also expanded. Over the past several years, numerous studies have explored topics such as motivation and perceived benefits (5), social connection and psychological well-being (6), physiological and activity effects (7, 8), social capital and happiness (9), as well as physical and cognitive health (2). Beyond psychological and social factors, recent studies have also focused on the physical demands of pickleball, which involves agility, muscular endurance, and balance (8, 10). This growing focus on physical demands has also drawn increased attention to potential injuries (11). Previous research has identified a range of injuries associated with pickleball, including muscle strains, joint sprains, and overuse injuries, which primarily affect the lower and upper extremities (12, 13). For example, Forrester (12), first identified 300 pickleball related injuries from the National Electronic Injury Surveillance System (NEISS) database maintained by the US Consumer Product Safety Commission from 2001 to 2017. The most common injuries were strains or sprains (28.7%) and fractures (27.7%), followed by contusions or abrasions (11.9%), lacerations (5.9%), and internal injuries (5.6%). Injuries were most frequently reported in the lower extremities (32.0%), followed by the upper extremities (25.4%), trunk (21.4%), and head/neck (16.9%). Weiss et al. (13) expanded on this research by analyzing injuries from the same NEISS database between 2010 and 2019, applying standardized terminology to address inconsistencies and misspellings identified in the original dataset. They reported similar findings, with variations in injury distribution percentages likely due to overlapping data from the same source.

Notably, pickleball plays involving multidirectional movement patterns, abrupt acceleration-deceleration, and repetitive swing action may impose cumulative stress on the musculoskeletal system (14, 15). These repetitive and multidirectional movements can result in cumulative mechanical loading, which may lead to overuse injuries that develop gradually and are not caused by a single traumatic event (15, 16). While acute injuries require immediate medical attention, less severe injuries such as minor, overuse or chronic injuries are more frequently managed in outpatient clinics or sports rehabilitation settings. As a result, less severe or non-acute injuries are not fully captured by surveillance systems, which in turn may contribute to the underestimation of these injuries in pickleball players, as seen in other racket sports (17, 18). Therefore, further research is needed to better understand the various aspects of pickleball related injuries beyond those observed in emergency room settings.

In addition, investigating the risk factors for injuries in pickleball could offer valuable insights into improving player safety. Although there are currently no published studies that explore this aspect specifically in pickleball, insights from research on racket and paddle sports can be highly informative (19). Factors such as skill level, playing hours, age, and gender are known to significantly influence injury risk in similar sports (12, 13, 20–26). For instance, studies have shown that professional players generally experience fewer injuries compared to their recreational counterparts (20, 21). Recreational players with lower technical proficiency tend to face an increased risk of injury (22, 23). Additionally, extended playing durations have been associated with a higher prevalence of injuries, as seen in tennis (22, 24, 25). Age is a well-documented risk factor for injuries in pickleball (12, 13, 26). Older adults are particularly susceptible, with the average age of injury reported as 63 years and 87.3% of injuries occurring in players aged 50 or older (12). Injury prevalence has been reported to increase progressively with age, particularly among those in their 60s and older, compared to younger counterparts (12, 13, 26). Among older adult players, injuries most commonly affect the lower extremities, particularly the knee and ankle, consistent with prior epidemiological findings (12, 13). These trends underscore the increased vulnerability of older populations to pickleball-related injuries. Given this elevated risk, age-specific injury prevention efforts are warranted to support safe participation and sustained engagement in the sport. In addition to age, gender may also influence injury risk in pickleball. A recent study by Kim et al. (27) reported that male players had a higher injury prevalence than female players. This difference may be partially explained by greater play intensity and physical demands among male players, who often use heavier paddles and cover more court space at a faster pace. Although research on pickleball related injuries is emerging, studies investigating specific risk factors such as age, gender, skill level, and total weekly play hours among South Korean players remain limited. This gap in the literature hinders the development of effective prevention strategies. Thus, through a cross-sectional survey design, the present study aimed to (1) examine the 12-month prevalence and characteristics of pickleball-related injuries—including body region, tissue type, and injury mechanism—and (2) identify demographic and play characteristic risk factors associated with injury occurrence. The findings can help inform targeted injury prevention programs and public health recommendations, ultimately supporting the safe growth of pickleball as a popular, health-promoting activity that fosters community and well-being.

## 2 Materials and methods

### 2.1 Participants

This study employed a cross-sectional survey method, with data collected during the championship, held in Cheongju, South Korea, in 2024. Researchers set up an on-site booth to recruit eligible participants. Inclusion criteria were (1) age 18 years or older, (2) participation in the tournament, and (3) ability to read and understand Korean language. Exclusion criteria were (1) pre-existing injuries unrelated to pickleball and (2) inability to complete the survey due to personal reasons. The questionnaire took approximately 10–15 min to complete. Completed surveys were returned immediately to the researchers on site. To ensure anonymity, no names or personally identifiable information were collected on the questionnaires. All respondents were provided with a small non-monetary incentive (two pairs of athletic socks) when completing the questionnaire. The University’s Institutional Review Board approved the study (SKKU 2024 04–055), and all participants provided informed written consent before taking the survey.

### 2.2 Questionnaire

The questionnaire was developed by a multidisciplinary team with experience in survey development, including medical doctors, certified athletic trainers, and researchers in sports science, clinical rehabilitation, and the social and behavioral sciences. Its structure and content were adapted from previous studies (27) conducted in recreational pickleball populations to ensure contextual relevance and applicability. It was designed to collect comprehensive participant information across several domains: demographics, play characteristics, and previous injury history. Demographic information included age and gender. The play characteristics encompassed participants’ self-reported skill level. This was assessed using a categorical scale based on the Korea Pickleball Association’s official classification system (28, 29), The scale includes: level 1.0–2.0 (just started, unfamiliar with rules), level 2.5 (some experience, short rallies possible), level 3.0 (consistent forehand, limited backhand), level 3.5 (intermediate control of both hands, basic dinking), level 4.0 (controlled dink and 3rd shot drop), level 4.5 (consistent aggressive dinking and swinging volleys), and level 5.0–5.5 + (*advanced control of all strokes*). This system adopts rating definitions from the US Pickleball Association (30) and includes discrete categories: under 3, 3, 3.5, 4, and over 4, along with a “Do not know” option for participants uncertain of their level. Additional questions included weekly play hours, frequency of play per week, and the length of their pickleball playing experience. Also, participants reported on their warm-up and cool-down routines by selecting the types of activities performed (e.g., stretching, walking, cycling, jogging, or drill practice), and the duration of these routines was categorized as <5 min, 5–10 min, 10–20 min, 20–30 min, or >30 min. Participants were also asked whether they had sustained any injuries while playing pickleball during the past 12 months. If they indicated a history of injuries, follow-up questions captured details about the affected body parts and body tissue. An anatomical body chart adapted from the standard Nordic Musculoskeletal Questionnaire (NMQ) was used to allow clearer visualization of the affected body regions (31). Further questions addressed the onset of injuries, which participants were asked to classify as either traumatic (resulting from a specific identifiable event) or overuse (resulting from gradual onset). In this study, overuse injuries were operationally defined as those with a gradual onset and no specific traumatic incident, based on participants’ self-reported experiences. Additional questions included duration of activity cessation due to injury, and changes in physical activity levels following injuries.

### 2.3 Statistical analysis

Descriptive statistics were performed for all variables, with continuous variables reported as means and standard deviations (SD) and with categorical variables presented as frequencies and percentages. We employed backward elimination and stepwise logistic regression analysis to identify significant predictors, ensuring it retains only statistically significant and relevant variables. The model included demographic factors (age and gender) and play characteristics (skill level, the length of playing experience, frequency of play per week, and weekly play hours). Additionally, odds ratios (OR) with 95% confidence intervals (CI) were calculated to quantify the strength of these associations. OR greater than 1 indicated higher odds of injury, whereas OR less than 1 indicated lower odds of injury associated with the predictor variable (32). Additionally, effect size strength was interpreted based on published interpretation guidelines (33). For odds ratios (ORs) greater than 1.0, values less than 1.5 were considered weak effects, approximately 3.47 as moderate effects, and approximately 6.71 as large effects. For ORs less than 1.0, inverse values (1/OR) were used for interpretation, with values greater than 0.60 as weak effects, approximately 0.29 as moderate effects, and approximately 0.15 as large effects. All statistical analyses were conducted using SPSS version 25, with a two-tailed significance level set at *p* < 0.05.

## 3 Results

### 3.1 Participants

Table 1 provides a descriptive summary of demographics and play characteristics for the 232 participants included in the study. The participants exhibited an age range from 23 to 71 years, with a mean age of 50.5 ± 12.2 years. The sample comprised 136 males (58.6%) and 96 females (41.4%). The majority of participants (61.2%) were aged 50 years or older, with the largest age group being those in their 50s (35.8%). Regarding skill level, the largest group (26.3%) of participants reported a level under 3, followed by 27.6% at level 3, 19.0% at level 3.5, 10.3% at level 4, and only 3.0% reported a level over 4. A small proportion (13.8%) responded “Do not know.” Specifically, male participants were more likely to report higher skill levels, with 18.5% at level 3 and 10.8% at level 3.5. In contrast, female participants were more concentrated in the lower categories, with 27.6% under level 3 and only 2.6% at level 4 or above. The participants reported length of playing experience for an average of 20.3 ± 15.9 months, with men and women showing similar levels of participation. On average, participants played 3.4 ± 1.4 times a week, with 29.7% reporting they played three times and 18.5% reporting four times. The average weekly play hours were 9.6 ± 8.2 h, with men averaging 9.3 h and women averaging 10.1 h per week. Most participants engaged in warm-up before play, with 95.6% of participants indicating that they performed warm-up routines. Among the warm-up types, stretching was the most reported (78.1%), followed by walking (7.9%), pickleball drill practice (6.0%), running (3.6%), cycling (2.4%), and other methods (2.0%). In terms of warm-up duration, nearly half of the participants (47.2%) dedicated between 5 to 10 min to warming up, while 31.1% reported warming up for less than 5 min. A smaller group (16.2%) extended this period to 10–20 min, and very few participants (5.5%) warmed up for over 20 min.

**Table 1:** Descriptive summary of demographics, playing characteristics, and self-reported injuries.

Characteristics			Mean±Standard deviation(Proportion, %)
Demographics			
Gender	Male (n = 136)	Female (n = 96)	Total (n = 232)
Age	49.8 ± 12.6 (58.6%)	51.5 ± 11.6 (41.4%)	50.5 ± 12.2 (100.0%)
20–29	25.6 ± 2.0 (5.6%)	26.1 ± 1.9 (3.9%)	25.8 ± 1.9 (9.5%)
30–39	33.6 ± 2.6 (8.2%)	36.0 ± 2.0 (2.2%)	34.1 ± 2.7 (10.4%)
40–49	45.5 ± 2.2 (8.6%)	46.4 ± 2.3 (10.3%)	46.0 ± 2.3 (18.9%)
50–59	54.3 ± 2.8 (21.6%)	54.7 ± 3.1 (14.2%)	54.4 ± 2.9 (35.8%)
60+	63.8 ± 2.7 (14.6%)	64.5 ± 1.9 (10.8%)	64.1 ± 2.4 (25.4%)
Play characteristics			
Skill Level	3.2 ± 1.4 (58.6%)	2.6 ± 1.2 (41.4%)	3.0 ± 1.3 (100.0%)
Do not know	5.2%	8.6%	13.8%
Under 3	13.4%	12.9%	26.3%
3	18.5%	9.1%	27.6%
3.5	10.8%	8.2%	19.0%
4	7.7%	2.6%	10.3%
Over 4	3.0%	0.0%	3.0%
Length of Playing Experience	20.2 ± 16.6 (58.6%)	20.6 ± 14.9 (41.4%)	20.3 ± 15.9 (100.0%)
Weekly Play Hours	9.3 ± 6.0 (58.6%)	10.1 ± 10.7 (41.4%)	9.6 ± 8.2 (100.0%)
Frequency of Play Per Week	3.3 ± 1.4 (58.6%)	3.5 ± 1.6 (41.4%)	3.4 ± 1.4 (100.0%)
Once a week	3.9%	3.0%	6.9%
Twice a week	14.7%	7.8%	22.5%
Three times a week	15.9%	13.8%	29.7%
Four times a week	11.6%	6.9%	18.5%
Five times a week	9.1%	5.6%	14.7%
Six times a week	2.6%	1.3%	3.9%
Every day	0.9%	3.0%	3.9%
Warm-up	(58.4%)	(41.6%)	(100.0%)
Yes	56.2%	39.4%	95.6%
No	2.2%	2.2%	4.4%
Types of Warm up	(59.9%)	(40.1%)	(100.0%)
Walking	5.5%	2.4%	7.9%
Running	2.8%	0.8%	3.6%
Cycling	2.0%	0.4%	2.4%
Stretching	44.8%	33.3%	78.1%
Drill practice	3.6%	2.4%	6.0%
Other	1.2%	0.8%	2.0%
Length of Warm-up	(59.0%)	(41.0%)	(100.0%)
Less than 5 min.	14.0%	17.1%	31.1%
5–10 min.	28.3%	18.9%	47.2%
10–20 min.	12.0%	4.1%	16.2%
20–30 min.	3.2%	0.9%	4.1%
More than 30 min.	1.4%	0.0%	1.4%
Self-reported injuries			
Injury Experience	(58.4%)	(41.6%)	(100.0%)
Yes	20.3%	13.9%	34.2%
No	38.1%	27.7%	65.8%
Playtime lost due to injury	(59.6%)	(40.4%)	(100.0%)
1–2 days	15.2%	7.6%	22.8%
Less than 1 week	13.9%	5.1%	19.0%
1–2 weeks	12.7%	7.6%	20.3%
2–4 weeks	12.7%	8.8%	21.5%
1 month or more	5.1%	11.3%	16.4%
Change in activity intensity	(58.5%)	(41.5%)	(100.0%)
Yes	32.9%	30.4%	63.3%
No	26.6%	10.1%	36.7%

### 3.2 Prevalence and characteristics of self-reported injuries

**Figure 1:**
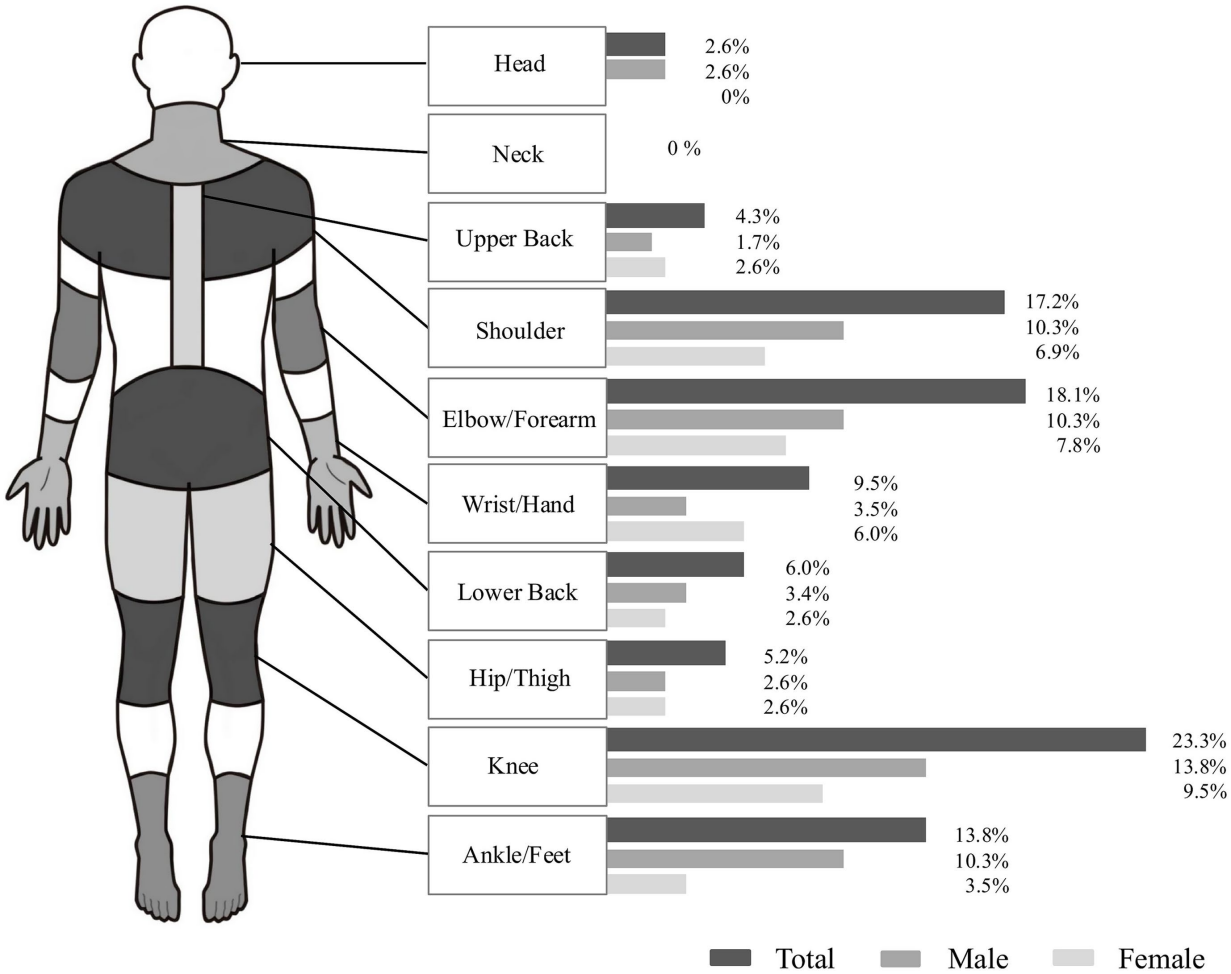
Self-reported injuries by body part from the 2024 Korea Open Pickleball Championship.

Seventy-nine participants (34.2%) reported experiencing an injury while playing pickleball over the past 12 months. Among these 79 participants, 62 (78%) identified their injuries as overuse injuries, while 17 (22%) indicated traumatic injuries. Figure 1 illustrates the distribution of injuries across anatomical regions. The majority of injuries affected the upper extremities (44.8%) and lower extremities (42.3%). Upper extremity injuries were more frequently reported, particularly in the elbow/forearm (18.1%), shoulder/arm (17.2%), and wrist/hand (9.5%). Among lower extremity injuries, the knee was the most frequently affected site (23.3%), followed by the ankle/foot (13.8%). Trunk injuries were less frequent, including the lower back (6.0%) and upper back (4.3%). Head and neck injuries were rare, reported in 2.6 and 0% of cases, respectively.

**Figure 2:**
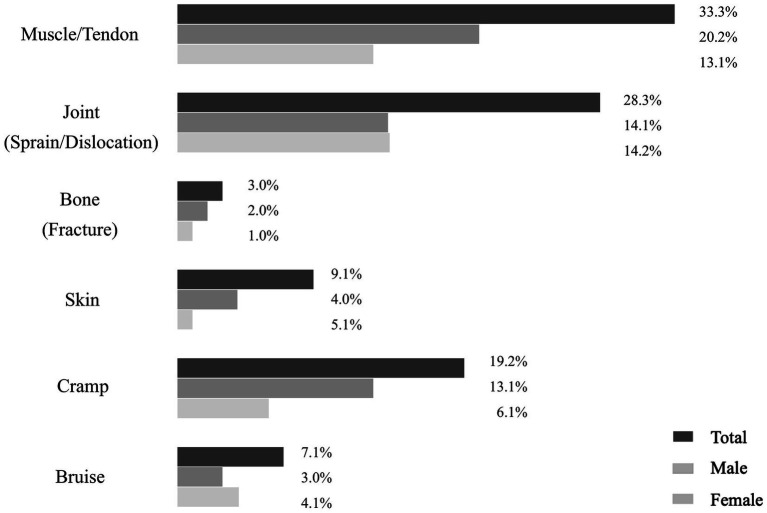
Self-reported injuries by tissue type at the 2024 Korea Open Pickleball Championship.

Regarding the type of body tissue, injuries primarily occurred in muscle/tendon (33.3%), joint sprains/dislocations (28.3%), and muscle spasms (19.2%), as shown in Figure 2. Most injuries (63.3%) led to a reduction in activity intensity and 20.3% reporting modifications in their skills or participation style to prevent further injuries. The severity of these injuries, as estimated by the self-reported duration of play cessation, varied: 22.8% of participants repored only 1–2 days off, 19.0% less than 1 week, 20.3% between one and 2 weeks, 21.5% required 2 to 4 weeks of absence, and 16.4% more than a month.

### 3.3 Risk factors associated with presence of injuries

Table 2 provides an overview of the backward stepwise logistic regression analyses undertaken to identify significant risk factors associated with pickleball injuries. The final model included weekly play hours and skill level, significantly enhancing the model’s explanatory power (χ^2^ [2] = 20.33, *p* < 0.01). The model’s adjusted R-square value was 0.124 (*p* < 0.01). This indicates that weekly play hours and skill level explained approximately 12.4% of the likelihood of injury. Skill level was a statistically significant but weak predictor of injury risk (B = −0.237, SE = 0.118, *p* = 0.044). The negative coefficient indicates that higher skill levels were associated with a lower likelihood of injury. Specifically, the odds ratio was 0.789; 95% CI: 0.624–0.992, suggesting that for each one unit increase in skill level, the odds of sustaining an injury decreased by approximately 21.1%. Similarly, weekly play hours showed a statistically significant but weak negative association with injury risk in the final model (B = −0.091, SE = 0.029, *p* = 0.001). The odds ratio for this factor was 0.913; 95% CI: 0.861–0.963, meaning that for each additional hour of weekly play, the odds of injury decreased by about 8.7%. In summary, higher skill level and greater weekly play hours were identified as the strongest predictors of reduced injury risk in our final model.

**Table 2:** Backward stepwise logistic regression analyses of injury risk factors.

Variables	χ^2^ (df)	Δχ^2^ (df)	B	SE B	Wald	df	*p*-value	Odds ratio	95% CI for odds ratio
Step 1: Full model									
Intercept	21.74 (6)*	-	1.793	0.825	4.727	1	0.030*	6.010	(1.240, 31.967)
Gender			−0.044	0.324	0.018	1	0.892	0.957	(0.507, 1.814)
Age			0.014	0.014	1.095	1	0.295	1.014	(0.988, 1.042)
Skill level			−0.208	0.141	2.153	1	0.142	0.813	(0.613, 1.072)
Length of playing experience			−0.002	0.012	0.043	1	0.836	0.998	(0.975, 1.021)
Frequency of play per week			−0.119	0.158	0.562	1	0.454	0.888	(0.657, 1.218)
Weekly play hours			−0.073	0.041	3.201	1	0.074	0.930	(0.854, 0.993)
Step 2: Length of playing experience removed									
Intercept	21.70 (5)*	0.04 (1)	1.832	0.805	5.180	1	0.023*	6.245	(1.339, 31.878)
Gender			−0.054	0.320	0.029	1	0.865	0.947	(0.506, 1.781)
Age			0.014	0.013	1.117	1	0.304	1.014	(0.988, 1.041)
Frequency of play per week			−0.120	0.158	0.576	1	0.449	0.887	(0.657, 1.216)
Weekly play hours			−0.072	0.041	3.188	1	0.074	0.930	(0.855, 0.993)
Skill level			−0.223	0.122	3.343	1	0.068	0.800	(0.629, 1.016)
Step 3: Gender removed									
Intercept	21.67 (4)*	0.03 (1)	1.806	0.790	5.221	1	0.022*	6.088	(1.343, 30.219)
Age			0.013	0.013	1.049	1	0.306	1.014	(0.988, 1.041)
Frequency of play per week			−0.123	0.157	0.614	1	0.433	0.884	(0.656, 1.209)
Weekly play hours			−0.072	0.040	3.163	1	0.075	0.931	(0.855, 0.993)
Skill level			−0.218	0.119	3.368	1	0.067	0.804	(0.635, 1.014)
Step 4: Frequency of play per week removed									
Intercept	21.07 (3)*	0.60 (1)	1.741	0.784	4.939	1	0.026*	5.704	(1.274, 27.896)
Age			0.011	0.013	0.550	1	0.389	1.011	(0.986, 1.037)
Weekly play hours			−0.095	0.029	10.568	1	0.001**	0.909	(0.856, 0.960)
Skill level			−0.224	0.119	3.558	1	0.059	0.799	(0.631, 1.008)
Step 5 (Final Model): Age removed									
Intercept	20.33 (2)**	0.74 (1)	2.297	0.460	24.984	1	<0.001**	9.943	(4.177, 25.469)
Skill level			−0.237	0.118	4.074	1	0.044*	0.789	(0.624, 0.992)
Weekly play hours			−0.091	0.029	10.112	1	0.001**	0.913	(0.861, 0.963)

*p < 0.05, **p < 0.01.

## 4 Discussion

### 4.1 Prevalence and characteristics of pickleball injuries

The present study found that 34.2% of the participants experienced an injury in the past 12 months with the majority affecting the upper extremities (44.8%), and lower extremities (42.3%). A small proportion of injuries also occurred in other regions, including the head (2.6%) and upper/lower back (10.3%). This distribution contrasts with previous research, which generally reports a higher proportion of lower extremity. For instance, Forrester (12) reported that 32.0% of pickleball injuries treated in emergency departments involved the lower extremities and 25.4% involved the upper extremities. Similarly, Weiss et al. (13) observed that 36.8% of pickleball-related injuries affected the lower extremities, while 28.7% involved the upper extremities. These results suggest that lower extremity injuries are generally more prevalent, likely due to the demands of rapid directional changes, lunging, and jumping (13). These trends are consistent with findings from other racket sports. For example, Changstrom et al. (26) reported that lower extremity injuries accounted for 34% of cases in racket and paddle sports, while upper extremity injuries constituted 25%. Similarly, tennis players reported a lower extremity injury prevalence of 41.7% and an upper extremity injury prevalence of 25.0%, while badminton players reported 44.4% lower extremity injuries and 24.3% upper extremity injuries (19, 20). However, our participants reported a higher proportion of upper extremity injuries, whereas the prevalence of lower extremity injuries was generally consistent with previous findings. One possible explanation for this discrepancy is that our sample consists of adult recreational players. Research suggests that recreational players—owing to less refined technique and slower reaction times—are more vulnerable to upper extremity injuries, whereas competitive athletes, who engage in more intense and dynamic movements, tend to sustain more lower extremity injuries. For instance, Jayanthi et al. (34) reported that among recreational tennis players, the elbow (20%) and shoulder (15%) were the most frequently injured sites, highlighting the influence of skill level on injury distribution. Similarly, a systematic review on occurrence of tennis injuries (25) found that 21 to 45% of injuries in adult recreational tennis players involved the upper extremities. This interpretation is further supported by a recent epidemiological study of older recreational pickleball players, which found that upper extremity injuries accounted for 44.7% of all reported injuries (27). Also, this discrepancy may stem from the overall skill level of the participants. Given that our participants had an average skill rating of approximately 3.0, classifying them as beginner to low-intermediate players, their limited technical proficiency, suboptimal stroke mechanics, and slower reaction times may have contributed to the increased incidence of upper extremity injuries. Overall, this distribution highlights a shift toward upper extremity injury predominance among recreational participants.

Regarding injury types, our study found that injuries primarily occurred in muscle/tendon (33.3%), joint sprains/dislocations (28.3%), and muscle spasms (19.2%). The predominance of muscle/tendon injuries may be attributed to the repetitive nature of pickleball. Repetitive swinging places mechanical stress on the elbow and shoulder, while sudden directional changes and pivoting increase strain on the knee and ankle (35). These biomechanical demands can overload both muscles and tendons. These results are consistent with previous pickleball research, where Weiss et al. (13) found that the most common pickleball injuries included strains or sprains (32.4%) and fractures (25.5%). Similarly, Changstrom et al. (26) observed that in racket and paddle sports, sprains/strains accounted for 39.5%, fractures/dislocations for 18.8%, and skin injuries for 12.1% among patients treated in US emergency departments, primarily recreational participants across a wide age range. Tennis players, particularly those at amateur and semi-elite levels, report even higher muscle/tendon injury rates, with 84.0–87.7% of injuries affecting muscles or tendons (20). Likewise, recreational level badminton players experience sprains/strains (45.2%), soft tissue injuries (16.5%), and fractures/dislocations (14.9%), while squash and racquetball players report sprains/strains (33.8%), skin-level injuries (17.4%), and soft tissue injuries (16.1%) (19). The similarities in injury types across these racket and paddle sports may represent similar biomechanical stresses over joints and muscles/tendons.

Furthermore, the high prevalence of overuse injuries (78%) compared to traumatic injuries (22%) observed in this study aligns with research on other racket sports. For instance, Colberg et al. (36) examined collegiate tennis players and reported that while all injuries sustained during match play were acute in nature, 69.6% of injuries sustained during training were gradual in onset, and 27.6% of players had at least one chronic condition during the season. Pluim et al. (37) conducted a prospective study of elite junior tennis players and found in another investigation that overuse injuries emerged as the most common health problem (47%), whereas acute injuries accounted for only 13%. Although these studies involved high-performance athletes, our findings among recreational pickleball players revealed similar overuse injury patterns, despite differences in participant characteristics. These results underscore the long-term physical demands of racket sports, where repetitive, high-impact movements contribute to musculoskeletal strain. In tennis and badminton, actions such as rapid directional changes, forceful swings, and prolonged rallies have been linked to chronic conditions like tendinopathies, stress fractures, and joint degeneration (38–40). Pickleball involves similar movement patterns, including frequent pivoting, lunging, and swinging, which may also contribute to the development of overuse injuries (13). However, this association is currently inferred from biomechanical similarities with other racket sports, as no prior studies have explicitly measured chronic injuries in pickleball. Therefore, while the possibility of such injuries remains speculative, it underscores the need for longitudinal research that tracks injuries across tournaments.

### 4.2 Play characteristics associated with pickleball injuries

Our logistic regression analysis indicated that skill level was significantly associated with pickleball injuries, suggesting that players with higher skill levels may experience fewer injuries. For every one-unit increase in skill level the odds of experiencing an injury decrease by approximately 21.1%. These findings appear to align with earlier studies on racket sports, which suggest that “beginner or recreational-level participants” often face elevated injury risks compared to more experienced or professional athletes. For example, Kekelekis et al. (41) report higher injury risks among lower-skilled, recreational racket sports players, potentially due to inadequate technique compared to professional level. Similar results have been noted by Rice (23) in an analysis of elite versus sub-elite junior tennis players. These results confirm that proficiency in technique and experience in pickleball and racket sports more broadly play a critical role in mitigating injury risks. As skill level increases through additional practice time, players often refine their movement patterns, adopt more effective preventive strategies, and improve overall technique factors shown to reduce overuse injuries in other racket sports (42–45).

Additionally, our logistic regression analysis identified weekly play hours as a significant predictor, indicating that more frequent play was associated with a lower likelihood of injury. For each additional hour of weekly play, the odds of injury decreased by about 8.7%. This inverse relationship may seem counterintuitive. Notably, a recent pickleball-specific study by Kim et al. (27) reported a positive association between weekly play hours and injury risk among older recreational players. This finding directly contrasts with our results and may reflect differences in participant age, baseline physical condition. Especially since numerous studies in racket sports have linked greater training volumes with increased injury risk. In addition, numerous studies in other racket sports players who train more than 3–6 h per week are at an elevated risk for overuse injuries, especially in the shoulder and elbow regions (21, 23, 24, 46). Also, Minghelli and Cadete (21) also found that tennis players who trained more than 4.5 h per week had twice the probability of experiencing an injury. These conflicting results between the current study and previous research may stem from the distinct biomechanical demands of pickleball. Unlike tennis or squash, where forceful overhead serves and powerful groundstrokes are more common, pickleball predominantly relies on underhand serves and a relatively slower ball speed (15, 47, 48). This likely results in lower peak external forces during gameplay, potentially reducing the cumulative stress on players’ joints. In addition, many pickleball participants, particularly older adults, may self-regulate their intensity (49, 50), taking more frequent rest periods or engaging in doubles play, further mitigating overuse injuries (10, 44). For instance, Webber et al. (10) examining singles and doubles pickleball in older adults found that singles were classified as moderate intensity 80.5% of the time (via Freedson accelerometer cut-points), whereas doubles were moderate intensity only 50.4% of the time, with nearly half (49.6%) categorized as light intensity activity. Notably, the same study revealed that 22.3% of total singles playtime and 21.3% of doubles playtime involved inactive periods (e.g., resting between rallies). This overall reduction in continuous exertion especially in doubles suggests that older adults can self-regulate their intensity by incorporating brief rest intervals, even when they log substantial weekly play hours. Consequently, the time spent at higher intensities may remain lower than expected, potentially explaining why greater weekly play hours are associated with decreased injury risk among participants. In other words, the more time spent playing pickleball, the greater the development of skill sets, which in turn leads to a reduced risk of injury. However, Given the observational nature of this study, future research should explore causal relationships between skill acquisition and injury prevention using longitudinal designs that incorporate both self-reported data and medical records to ensure more accurate and objective outcome assessment. Additionally, biomechanical studies are needed to objectively evaluate joint stress, movement patterns, and kinetic loads associated with different levels of pickleball play.

### 4.3 Factors not associated with pickleball injuries

Demographic factors such as age and gender are often reported as critical influences on injury risk in pickleball and similar sports (12, 13, 16, 26). However, our analysis did not confirm a significant association. Pluim et al. (25) observed that in tennis, injury risk gradually increases with sex, age and Weiss et al. (13) reported that the average age of injury in pickleball was 63 years, with senior male players significantly more likely than females to suffer strains or sprains, particularly in the lower leg. Male tennis players also showed a higher likelihood of injuries compared to females (25). The discrepancies between our findings and previous studies may stem from differences in sample composition. In our study, the mean age was 50.5 years, and participants aged 60 and older made up only 25.4% of the sample, potentially reducing the influence of age on injury prevalence. Additionally, our study included chronic injuries that may have influenced non-significant findings, while prior research predominantly focused on acute injuries reported in emergency settings. Thus, future studies should address these differences by incorporating broader age distributions and distinguishing between acute and chronic injuries to provide a more comprehensive understanding of injury patterns in pickleball.

While length of playing experience was not significantly associated with injury prevalence in this study, this non-significance may be attributed to the relatively short average playing duration among participants. Previous research in paddle sports has consistently shown that players with limited experience (<5 years) are more likely to sustain injuries, as more experienced players tend to benefit from improved technique and physical adaptability (46). Given that pickleball remains relatively new in South Korea, participants in our study reported an average playing experience of 20.3 months, compared to 43.8 months in the United States (5). Consequently, many Korean players have not yet accumulated enough experience for its impact on injury risk to be fully observable.

Future research should investigate how prolonged experience influences injury patterns in pickleball as the sport grows, with a focus on comparing injury risks among groups categorized based on accumulated playing experience.

Similarly, frequency of play per week was also not significantly associated with injury prevalence in this study. This finding may be explained by our sample’s composition, which primarily included recreational players with relatively low play intensity and competitive demands, unlike professional or highly trained athletes examined in other studies (51–54). This contrasts with findings with Rangasamy et al. (55) who reported that participants playing more than 3 days a week had 2.21 times greater injury risk compared to those who played less frequently. This suggests that our participants may not have accumulated enough total sessions or training load to replicate the elevated risks observed in previous studies.

## 5 Clinical implications

The findings from this study have important implications for clinical practice and player safety in pickleball. Firstly, the significant association between higher skill levels and reduced injury risk highlights the value of structured, progressive skill development programs. Training should focus on improving technical proficiency, particularly for beginner and recreational players, to mitigate the risks associated with improper technique and poor movement patterns. Alongside technical training, providing basic injury prevention education may help promote safer participation, particularly for recreational players. Additionally, the negative relationship between weekly play hours and injury risk suggests that with progressive practice, the mechanical stress on muscles and joints is likely diminished, offering further protection against overuse injuries. It is possible that increased skill proficiency, when developed alongside greater play volume, may contribute to more efficient movement patterns and reduced injury risk. Although this relationship remains speculative due to the cross-sectional nature of our study, future research employing longitudinal or interventional designs may help clarify whether progressive play exposure contributes to injury mitigation through skill development. For older adults, encouraging doubles play over singles may serve as an effective strategy to reduce cumulative physical stress and minimize the risk of overuse injuries, particularly in the upper and lower extremities. In this regard, these findings suggest that clinicians and coaches should promote progressive skill development and encourage consistent play exposure to reduce injury risk among recreational players.

## 6 Limitations and recommendations for future research

This study has several limitations. First, reliance on self-reported data introduces the possibility of recall bias, as participants may have inaccurately recalled the type or timing over the 12-month period. This may have affected the reported prevalence and characteristics of injuries in the retrospective survey data. Given the challenges in conducting prospective studies, the use of self-report, the cross-sectional design was necessary to enable the collection of preliminary information that offers an initial snapshot of pickleball injury patterns. Future research employing objective data sources, such as medical records, prospective injury surveillance or mobile activity logs, would yield more accurate estimates of injury incidence and severity. Second limitation is that Body Mass Index (BMI) was not measured in this study due to privacy concerns, despite evidence suggesting that body composition can influence injury risk in racket and paddle sports. Some participants may have been uncomfortable disclosing sensitive information, resulting in limited anthropometric data. Collecting self-reported height and weight through anonymous surveys could offer a practical way to assess BMI while minimizing participant discomfort as these values are generally well recalled. This could enable a better understanding of how body composition influences injury risk. Third, the generalizability of our findings is limited by the sampling context and participant characteristics. Data were collected from a single tournament, and most participants were recreational players with relatively short playing experience. As a result, caution is warranted when applying these findings to more experienced or competitive populations, or when interpreting them as specific to pickleball alone. Future study should aim to include a more diverse population to improve external validity. Lastly, this study did not account for several additional factors that may influence injury risk. For example, sudden increases in training load are known to elevate the risk of overuse injuries (56), and decreased recovery such as rest and cool-down has also been associated with increased injury susceptibility (57). Additionally, the omission of participants’ participation in other physical activities and play on various surface (e.g., hard court, wooden court, and clay court) may have introduced unmeasured confounding, potentially affecting the interpretation of injury patterns specific to pickleball. Future research should incorporate these variables to better understand their role in the development and mitigation of injuries among pickleball players.

## 7 Conclusion

This descriptive study provides preliminary data on the injury rate among recreational pickleball players in South Korea. The majority of participants were middle-aged or older adults, primarily engaged in recreational play, with a relatively low average skill level and short playing experience. The current study found that 34.2% of the participants experienced injuries in the past 12 months. Injuries most frequently affected the elbow and shoulder for upper extremities, and in the knee and ankle for lower extremities. In terms of tissue type, muscle/tendon injuries were the most frequent, followed by joint-related injuries. Also, further logistic regression analyses discovered that longer weekly play hours and higher skill level were significantly associated with a lower likelihood of injury. In contrast, gender, age, length of playing experience, and frequency of play per week were not associated with pickleball injuries. These findings provide insights into developing targeted strategies to manage and prevent pickleball injuries, particularly as the sport’s popularity continues to grow and more individuals engage in regular play (15, 35, 40). Future research is warranted to validate these findings across broader age groups and competitive levels, and to identify additional contributing factors that may influence injury risk in pickleball players.

## Data Availability

The raw data supporting the conclusions of this article will be made available by the authors, without undue reservation.
